# VIS – A database on the distribution of fishes in inland and estuarine waters in Flanders, Belgium

**DOI:** 10.3897/zookeys.475.8556

**Published:** 2015-01-22

**Authors:** Dimitri Brosens, Jan Breine, Gerlinde Van Thuyne, Claude Belpaire, Peter Desmet, Hugo Verreycken

**Affiliations:** 1 Research Institute for Nature and Forest (INBO), Kliniekstraat 25, 1070, Brussels, Belgium; 2 Research Institute for Nature and Forest (INBO), Duboislaan 14, 1560, Groenendaal, Belgium

**Keywords:** Ecosystem functioning, fish-based index of biotic integrity, fish distribution, freshwater, brackish water, estuary, LifeWatch, open data, occurrence, observation, River Scheldt, River Yser, River Meuse

## Abstract

The Research Institute for Nature and Forest (INBO) has been performing standardized fish stock assessments in Flanders, Belgium. This Flemish Fish Monitoring Network aims to assess fish populations in public waters at regular time intervals in both inland waters and estuaries. This monitoring was set up in support of the Water Framework Directive, the Habitat Directive, the Eel Regulation, the Red List of fishes, fish stock management, biodiversity research, and to assess the colonization and spreading of non-native fish species. The collected data are consolidated in the Fish Information System or VIS. From VIS, the occurrence data are now published at the INBO IPT as two datasets: ‘VIS - Fishes in inland waters in Flanders, Belgium’ and ‘VIS - Fishes in estuarine waters in Flanders, Belgium’. Together these datasets represent a complete overview of the distribution and abundance of fish species pertaining in Flanders from late 1992 to the end of 2012. This data paper discusses both datasets together, as both have a similar methodology and structure. The inland waters dataset contains over 350,000 fish observations, sampled between 1992 and 2012 from over 2,000 locations in inland rivers, streams, canals, and enclosed waters in Flanders. The dataset includes 64 fish species, as well as a number of non-target species (mainly crustaceans). The estuarine waters dataset contains over 44,000 fish observations, sampled between 1995 and 2012 from almost 50 locations in the estuaries of the rivers Yser and Scheldt (“Zeeschelde”), including two sampling sites in the Netherlands. The dataset includes 69 fish species and a number of non-target crustacean species. To foster broad and collaborative use, the data are dedicated to the public domain under a Creative Commons Zero waiver and reference the INBO norms for data use.

## Data published through

The occurrence datasets are available at:

**VIS - Fishes in inland waters in Flanders, Belgium**

Source: http://dataset.inbo.be/vis-inland-occurrences

GBIF: http://www.gbif.org/dataset/823dc56e-f987-495c-98bf-43318719e30f

**VIS - Fishes in estuarine waters in Flanders, Belgium**

Source: http://dataset.inbo.be/vis-estuarine-occurrences

GBIF: http://www.gbif.org/dataset/274a36be-0626-41c1-a757-3064e05811a4

Reports (only in Dutch) can be generated or downloaded from: http://vis.milieuinfo.be/publicaties/rapporten-afvissingen

## Rationale

**Figure 1.**
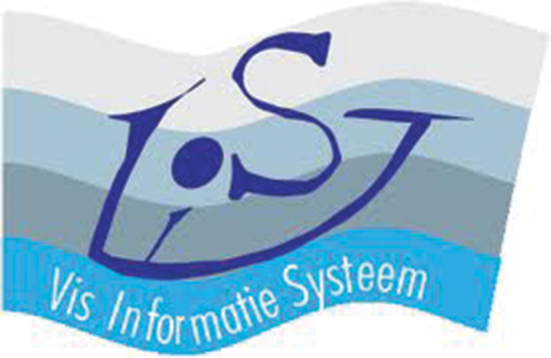
The logo of VIS.

The Fish Information System or VIS (Figure 1) is a database created by the Research Institute for Nature and Forest (INBO) which is used to monitor the status of fishes and their habitats in Flanders, Belgium and to calculate the biotic integrity (Karr 1981, Belpaire et al. 2000, Breine et al. 2004, 2007, 2010) of fish assemblages. It contains data regarding occurrences, individual morphometrics, stocks, pollutants, indices, and non-native fish species. Sampling has been going on since 1992, the database model was designed in 1994 (Verbiest et al. 1994), the first database developed in 1996 (Verbiest et al. 1996), and the consolidated database set up in 2001. VIS is used for supporting NATURA 2000, an ecological network of protected areas in Europe and to calculate the EQR (Ecological Quality Ratio) in the framework of the EU Water Directive (Directive 2000/60/EC). Further, the database provides updated information for Flemish Red Lists of fishes and lampreys (Verreycken et al. 2014) and on the distribution status of non-native and invasive fish species. The data are also crucial in fish stock management and for reporting on the status of the European eel stock as required by the Eel Regulation (Council Regulation (EC) N° 1100/2007).

## Taxonomic coverage

The inland waters dataset contains 64 fish species reported from Flemish enclosed waters and watercourses, as well as a number of non-target species (mainly crustaceans). This dataset also includes a number of typical brackish water fish species which sometimes can be found in inland water sites in proximity to the sea and/or behind the sluice gates. The class of Actinopterygii is best represented (63 species), along with one Petromyzontida (*Lampetra planeri*) and 7 crustaceans from the order Decapoda.

The estuarine waters dataset contains 69 fish species found in the estuaries of the River Yser and the River Scheldt, as well as 9 non-target crustacean species. The class of Actinopterygii is most represented (67 species), along with two Petromyzontida. All the crustaceans in this dataset are from the order of the Decapoda.

**Figure 2.**
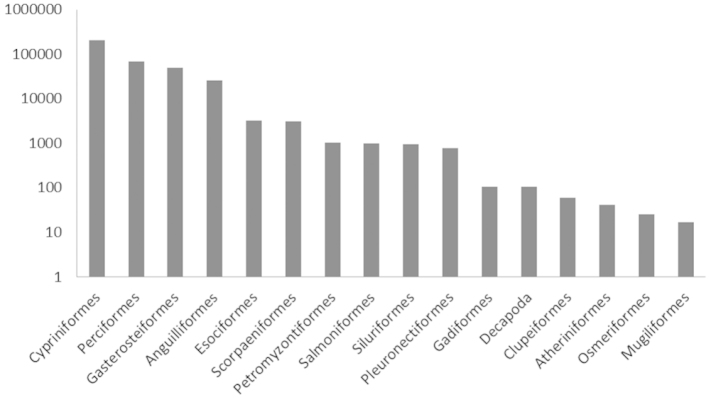
Distribution of all occurrences in the inland waters dataset by taxonomic order. Orders are ordered by number of occurrences, occurrences are displayed on a logarithmic scale.

**Figure 3.**
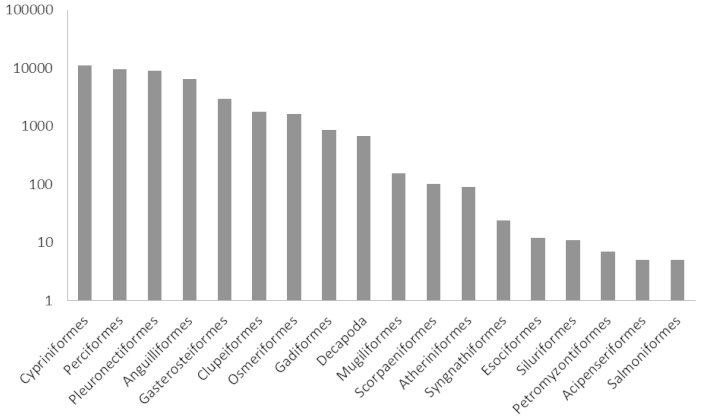
Distribution of all occurrences in the estuarine waters dataset by taxonomic order. Orders are ordered by number of occurrences, occurrences are displayed on a logarithmic scale.

In Figures 2 and 3 the distribution of occurrences by taxonomic order in the inland and estuarine waters dataset is shown.

## Taxonomic ranks for inland waters

**Kingdom:** Animalia

**Class:** Actinopterygii, **Orders:** Mugiliformes, Osmeriformes, Atheriniformes, Clupeiformes, Gadiformes, Pleuronectiformes, Siluriformes, Salmoniformes, Scorpaeniformes, Esociformes, Anguilliformes, Gasterosteiformes, Perciformes, Cypriniformes, **Families:** Anguillidae, Atherinidae, Centrarchidae, Clariidae, Clupeidae, Cobitidae, Cottidae, Cyprinidae, Esocidae, Gadidae, Gasterosteidae, Gobiidae, Ictaluridae, Lotidae, Moronidae, Mugilidae, Nemacheilidae, Osmeridae, Percidae, Petromyzontidae, Pleuronectidae, Salmonidae, Scophthalmidae, Siluridae, Soleidae, Umbridae

**Class:** Petromyzontida, **Order:** Petromyzontiformes, **Family:** Petromyzontidae

**Class:** Malacostraca, **Order:** Decapoda, **Families:** Atyidae, Cambaridae, Palaemonidae, Varunidae

## Taxonomic ranks for estuarine waters

**Kingdom:** Animalia

**Class:** Actinopterygii, **Orders:** Acipenseriformes, Anguilliformes, Atheriniformes, Clupeiformes, Cypriniformes, Esociformes, Gadiformes, Gasterosteiformes, Mugiliformes, Osmeriformes, Perciformes, Pleuronectiformes, Salmoniformes, Scorpaeniformes, Siluriformes, Syngnathiformes, **Families:** Acipenseridae, Agonidae, Ammodytidae, Anguillidae, Atherinidae, Blenniidae, Callionymidae, Carangidae, Centrachidae, Clupeidae, Cottidae, Cyprinidae, Esocidae, Gadidae, Gasterosteidae, Gobiidae, Liparidae, Lotidae, Moronidae, Mugilidae, Mullidae, Osmeridae, Percidae, Pholidae, Pleuronectidae, Salmonidae, Scophthalmidae, Siluridae, Soleidae, Syngnathidae, Trachinidae, Triglidae, Zoarcidae

**Class:** Petromyzontida, **Order:** Petromyzontiformes, **Family:** Petromyzontidae

**Class:** Malacostraca, **Order:** Decapoda, **Families:** Cambaridae, Cancridae, Crangonidae, Paguridae, Palaemonidae, Polybiidae, Portunidae, Varunidae

## Geographic coverage

### Flanders

**Figure 4.**
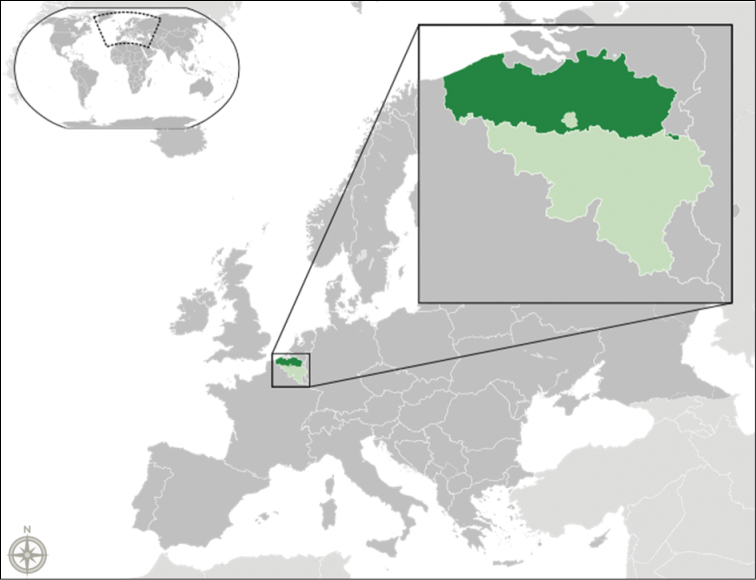
Flanders is an administrative region of Belgium, located in the centre of Western Europe. Image by Alphatron derived from Blank_map_of_Europe.svg, licensed under CC BY-SA 3.0.

Flanders is one of the three administrative regions in the country of Belgium, located in the centre of Western Europe (Figure 4). The Flemish region is situated in the north of the country and covers an area of 13,522 km² (44,29% of Belgium). Belgian has a temperate maritime climate that is influenced by the North Sea and the Atlantic Ocean with substantial precipitation in all seasons. The summers are moderate and the winters are mild. The two main geographical regions of Flanders are the coastal plain in the North-West and the Central plain, further inland. With 470 inhabitants/km², Flanders is one of the most densely populated areas of Europe. The three major rivers are the River Yser, the River Scheldt, and the River Meuse. All rivers in Flanders flow into the North Sea, but only the River Yser drains directly into the sea within the jurisdiction of Flanders.

### Inland waters

**Figure 5.**
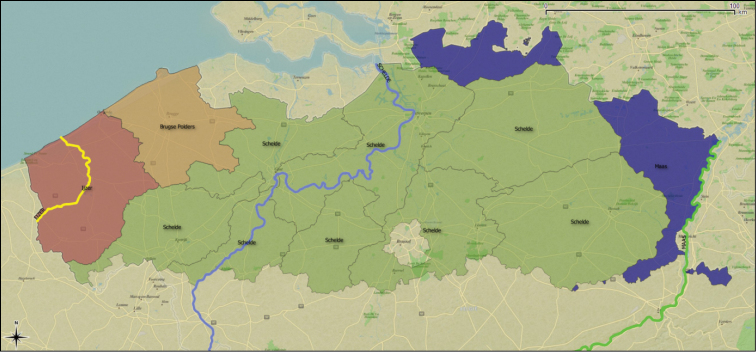
Drainages of the Rivers Yser (yellow in west), Scheldt (blue), and Meuse (green in east) are divided in 11 Flemish subbasins. The “Brugse polders” area drains directly to the sea. Image created in QGIS, basemap by Apple iPhoto map.

The inland waters dataset comprises enclosed waters, including cut off river arms, gravel pits, ponds, natural lakes, and artificial lakes; and riverine habitats, including head streams, tributaries, and canals part of the drainage basins of the rivers Yser, Scheldt and Meuse (Figure 5). These three drainage basins are divided into eleven Flemish river catchments, which are divided into 102 subbasins. Overall, there are 48 unique enclosed waters sampled at 792 locations and 419 streams and rivers sampled at 1,452 locations.

### Estuarine waters

The estuarine waters dataset comprises the estuaries of the River Scheldt, including tidal parts of the rivers Rupel, Durme, Zenne, Dijle and Grote Nete, and the River Yser.

**Figure 6.**
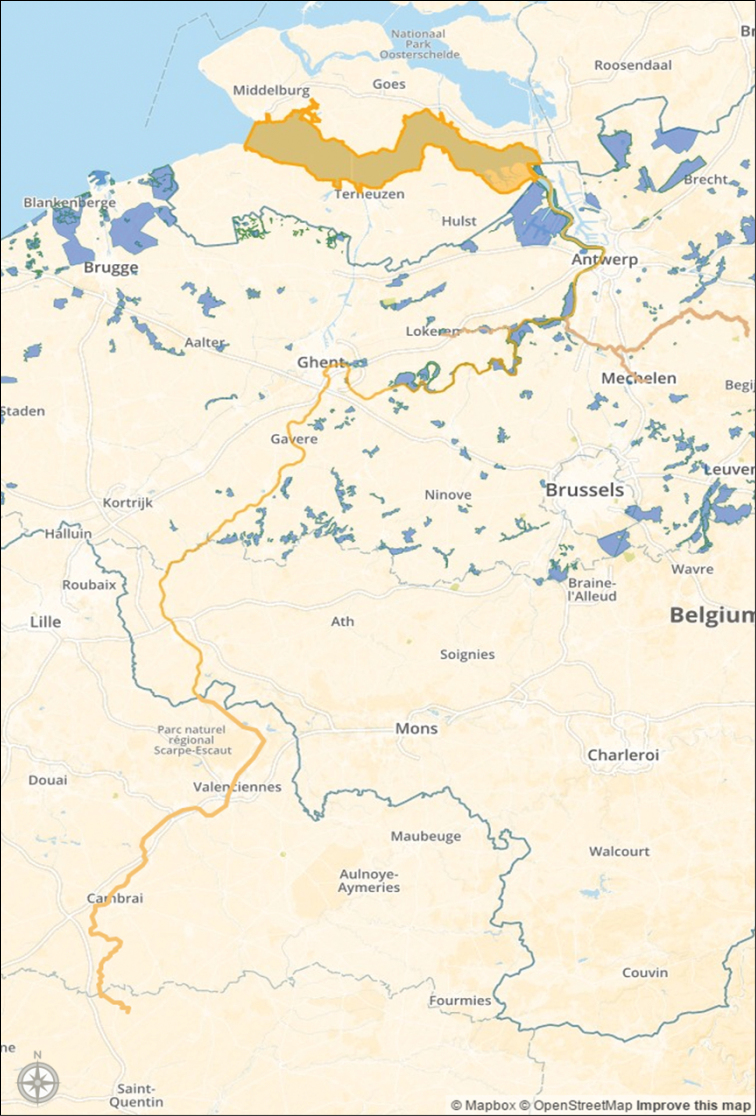
The river Scheldt (orange), from source to river mouth. Image created in Mapbox, basemap by OpenStreetMap contributors.

**River Scheldt.** The River Scheldt is a 435 km long lowland river originating on the plateau of Saint-Quentin (Figure 6) near Gouy, a small town in the French department of Aisne. The river enters Belgium close to Tournai. Then the river turns east, in the direction of Antwerp. After crossing the city of Antwerp, the Scheldt enters the Netherlands where it ends in the North Sea near Vlissingen. The tidal influence extends much further land inward than the freshwater-saltwater boundary. As a result, an extensive freshwater region under tidal influence is present. The tidal activity goes as far as Ghent, 160 km from the river mouth, where the tide is stopped by sluices. In the Zeeschelde (the Belgian part of the estuary), three zones are distinguished following the Venice system (1959): a mesohaline zone (5–18 g salt/kg) between Zandvliet and Antwerp, an oligohaline zone (0.5–5 g salt/kg) between Antwerp and Temse, including the Rupel tributary, and a tidal freshwater zone till Ghent including the Durme tributary. The marshes and mudflats create a valuable landscape for biodiversity.

**Figure 7.**
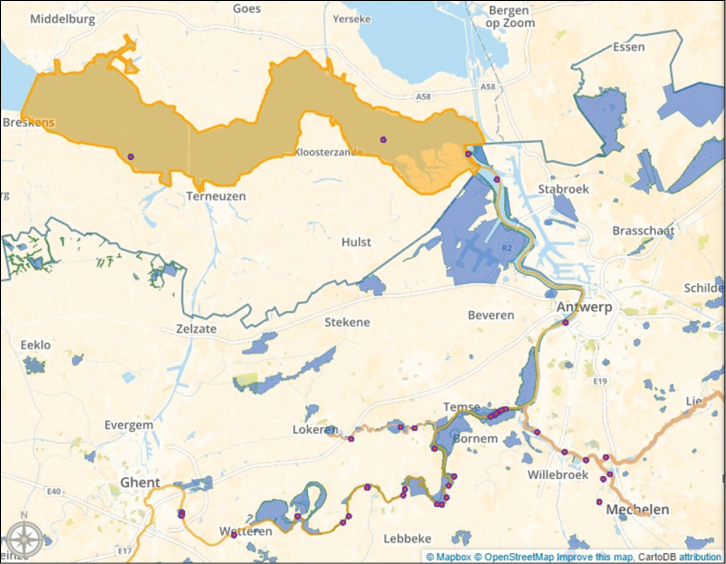
The Scheldt estuary, with sampling locations (pink points) and Natura 2000 areas in Flanders (blue areas). Image created in CartoDB and Mapbox, basemap by OpenStreetMap contributors.

The Scheldt estuary is one of the last natural deltas in Western Europe and many areas near its riverbanks are marked as Natura 2000 areas (Figure 7). Especially its freshwater estuary areas are unique.

**Figure 8.**
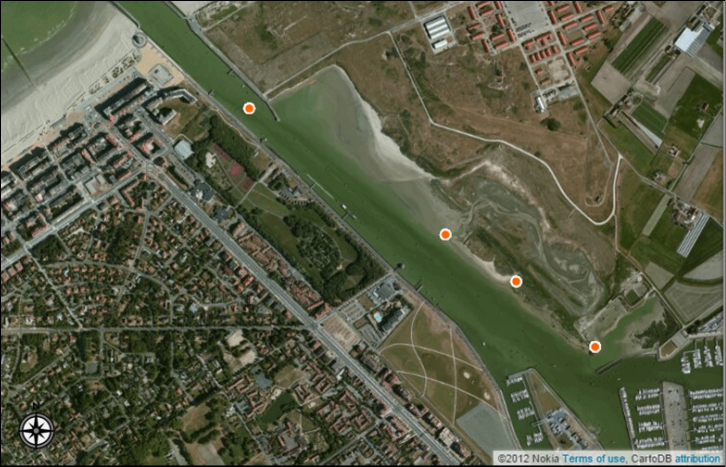
The sampling locations (orange points) in the Yser estuary. Image created in CartoDB, basemap by Nokia Satellite maps.

**River Yser.** The River Yser is a 78 km long river originating in Kassel, located in French Flanders. It enters Belgium in the province of West Flanders and drains into the sea near the town of Nieuwpoort. Sea and fresh water meet in an estuary, resulting in 130 hectares of tidal mud flats, salt marshes, dunes, beaches, and polders (Figure 8).

### Bounding box for covered area

Flanders: 50.68 to 51.51 latitude, 2.54 to 5.92 longitude.

Temporal coverage:

Inland waters: 1992-12-15 – 2012-11-27

Estuarine waters: 1995-04-01 – 2012-11-27

## Dataset

### Dataset description

The occurrence data from the VIS database are extracted, standardized, and published as two separate Darwin Core Archives: one for inland waters and one for estuarine waters. The main rationale behind this is that both datasets cover different habitats, differ in sampling strategies and methods, and are curated by different researchers. Nevertheless the data model used for inland waters and estuarine waters is identical and can be easily merged: together these datasets represent a complete overview of fish distribution in Flanders from late 1992 to the end of 2012. In 2013 a new set of sampling locations was defined and the data collected since then is currently only available upon request.

**Figure 9.**
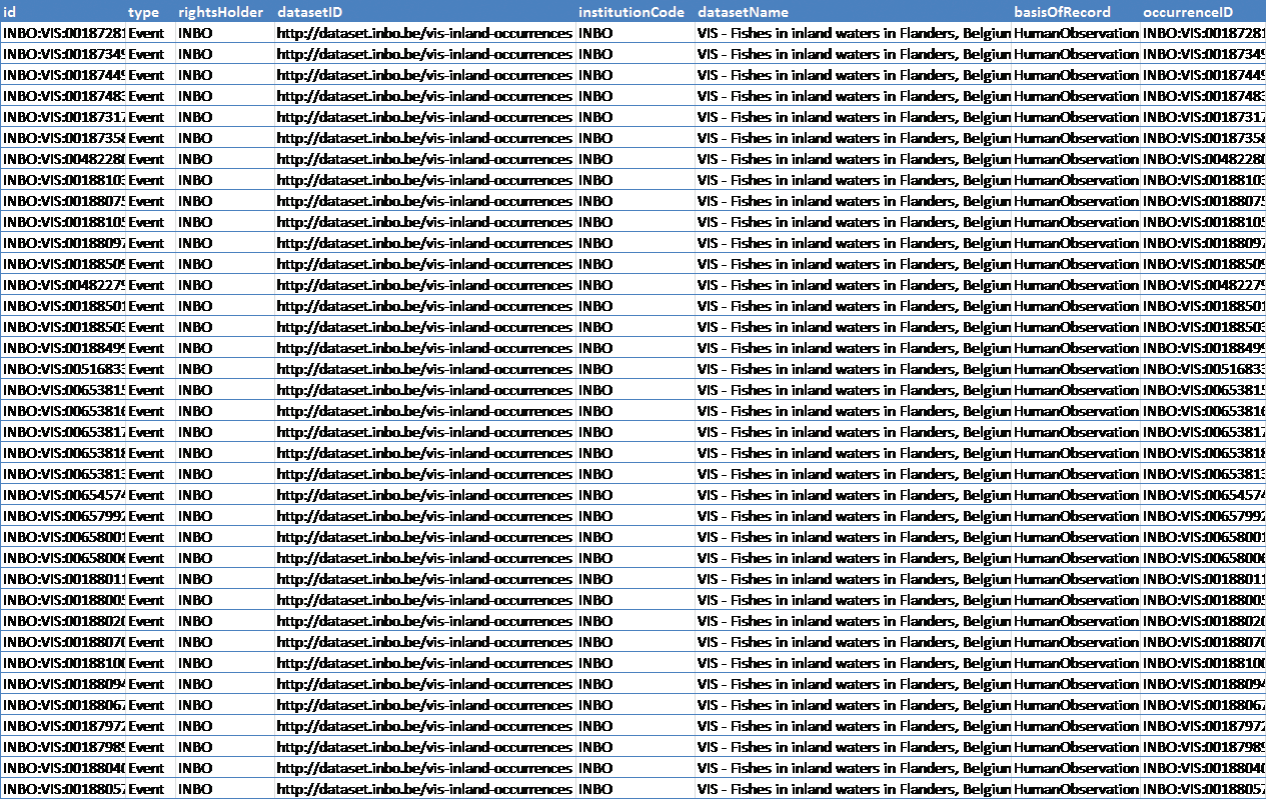
Preview of the Darwin Core SQL view of the inland waters dataset.

The data are standardized to Darwin Core (Wieczorek et al. 2012) with a custom SQL view (Figure 9) on the original VIS database and then published making use of the GBIF Integrated Publishing Toolkit (Robertson et al. 2014) instance at the INBO (http://data.inbo.be/ipt). The Darwin Core terms (http://rs.tdwg.org/dwc/terms/) in the dataset at the time of publication are:

occurrenceID, type, language, rights, rightsholder, accessRights, datasetID, institutionCode, datasetName, ownerInstitutionCode, basisOfRecord, informationWithheld, recordedBy, individualCount, samplingProtocol, samplingEffort, eventDate, habitat, locationID, continent, waterBody, countryCode, verbatimLocality, verbatimLatitude, verbatimLongitude, verbatimCoordinateSystem, verbatimSRS, decimalLatitude, decimalLongitude, geodeticDatum, coordinateUncertaintyInMeters, identifiedBy, scientificName, kingdom, taxonRank, scientificNameAuthorship, vernacularName, and nomenclaturalCode.

The data are dedicated to the public domain under Creative Commons Zero waiver. It would be much appreciated if you follow our norms for data use and notify the corresponding authors of the respective dataset if you use the data, especially for research purposes.

### Inland waters dataset

**Object name:** VIS - Fishes in inland waters in Flanders, Belgium

**Character encoding:** UTF-8

**Format name:** Darwin Core Archive format

**Format version:** 1.0

**Distribution:** http://dataset.inbo.be/vis-inland-occurrences

**Publication date of data:** 2013-12-20

**Language:** English

**Licenses of use:** http://creativecommons.org/publicdomain/zero/1.0/ & https://github.com/LifeWatchINBO/norms-for-data-use

**Metadata language:** English

**Date of metadata creation:** 2013-12-20

**Hierarchy level:** Dataset

### Estuarine waters dataset

**Object name:** VIS - Fishes in estuarine waters in Flanders, Belgium

**Character encoding:** UTF-8

**Format name:** Darwin Core Archive format

**Format version:** 1.0

**Distribution:** http://dataset.inbo.be/vis-estuarine-occurrences

**Publication date of data:** 2014-04-02

**Language:** English

**Licenses of use:** http://creativecommons.org/publicdomain/zero/1.0/ & https://github.com/LifeWatchINBO/norms-for-data-use

**Metadata language:** English

**Date of metadata creation:** 2014-04-02

**Hierarchy level:** Dataset

### Additional information

Length and weight measurement data of the individual fishes, absence information, occurrence data since 2013, as well as abiotic data of the sampling points (pH, temperature, etc.) are not included in the Darwin Core Archives and are available upon request.

## Methodology

### Study extent description

**Figure 10.**
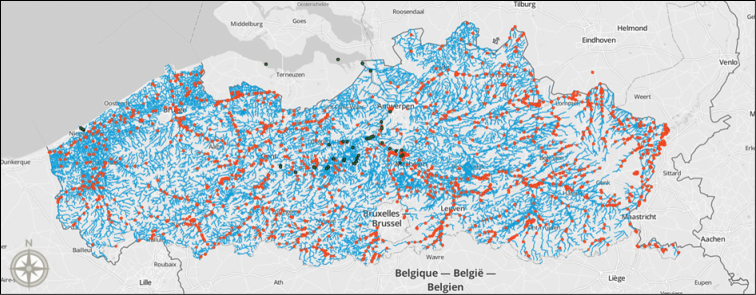
Map of all sampling locations in VIS. Orange points represent inland waters, green points represent estuarine waters. Image created in CartoDB and Mapbox, basemap by OpenStreetMap contributors.

Over 2,000 locations in estuaries, inland rivers, streams, canals, and enclosed waters in Flanders, Belgium have been sampled, from March to November, since 1992 (Figure 10). In 2001, these locations were consolidated in a monitoring network (“VISmeetnet”) of 900 sampling points. Four locations in the Yser estuary and 43 locations in the Scheldt were sampled since 1995. While the Yser estuary only covers a small geographical area, the Scheldt estuary is with 33,000 hectares one of the largest estuaries in Europe. It is also one of the few remaining European estuaries that includes the entire gradient from fresh to saltwater tidal areas (Van den Bergh et al. 2009). The 43 sampling locations in the Scheldt estuary are mainly located in the River Scheldt, but also in the Rivers Durme, Rupel, Dijle, Zenne and Nete.

The geographic coordinates in both datasets are those of the defined sampling locations (dwc:locationID). However, as these coordinates are not always exact the actual coordinates of the catch, which may be located further up- or downriver, the coordinate uncertainty (dwc:coordinateUncertaintyInMeters) has been set to 250 meter.

### Sampling description

In inland waters, standardized sampling methods were used as described in Belpaire et al. (2000) and Van Thuyne (2010) and are specified in the dataset as dwc:samplingProtocol. Per water body, the same method was used for each sampling event. The default method is electric fishing, but additional techniques such as gill nets, fykes, and seine netting (variable sizes) were used as well. Electric fishing was carried out using a 5 kW generator with an adjustable output voltage of 300–500 V and a pulse frequency of 480 Hz. The number of electric fishing devices and hand-held anodes used depends on the river width (Belpaire et al. 2000). In riverine environments, electric fishing was carried out on both riverbanks in upstream direction. All fishes were identified to species level, counted, and their length and weight was measured.

The default method used in estuarine waters is paired fyke netting, which has been intercalibrated by the North East Atlantic Calibration Group, but additional techniques such as anchor netting, seine netting, pound netting, electric fishing, and eel fyke netting were used as well (Breine et al. 2011). All fishes were identified to species level, counted and their length and weight was measured.

Fyke nets are relatively unselective fishing gear catching demersal and pelagic species (Hamerlynck and Hostens 1994) and also they are easy to install in a great variety of habitat types. As few studies compare fyke catches with other gear (e.g. Hinz 1989, Thiel and Potter 2001), we compared presence/absence data obtained with fyke nets with presence/absence data of fish impinged at cooling-water filter screens of the nuclear power plant of Doel situated in the study area. The data was collected in the same period between 1995 and 1998. During this period we collected the same species with both survey methods but the species richness per day per fyke net was generally higher than that obtained on the filter screens (Breine et al. 2007). In addition preliminary results from a gear intercalibration exercise in different estuaries in Ireland (Whyte et al. 2007) indicated that for species diversity, the results of fyke net catches are comparable to those obtained with other gear (beach seine, beam trawl, otter trawl).

### Quality control description

Strict field protocols where used. The Manual for Application of the European Fish Index (EFI) (Fame consortium 2004) served as a guideline for electrofishing and was used in support of the EU water framework directive.

Users of the data can comment on the inland waters and estuarine waters dataset at https://github.com/LifeWatchINBO/vis-inland-occurrences and https://github.com/LifeWatchINBO/vis-estuarine-occurrences respectively.

### Method step description

**Method step description:** 

Water type	Method	Effort
Running freshwaters (width: 1.5 m, depth: < 1.30 m)	electrofishing with 1 anode by wading	100 m
Running freshwaters (width: 6 m, depth: < 1.30 m)	electrofishing with 2 anodes by wading	100 m
Running freshwaters (width: > 6 m, depth: < 1.30 m)	electrofishing with 2 anodes by wading	250 m with 1 anode on each riverbank, 2 m from bank
Running freshwaters, streaming rivers (width: > 6 m, depth: > 1.30 m)	electrofishing with 2 anodes by boat	250 m with 2 anodes on each riverbank, 2 m from bank
Canals, slowly running rivers (width: > 6 m, depth: > 1.30 m)	electrofishing with 2 anodes by boat AND 2 fykes	250 m with 2 anodes on each riverbank, 2 m from bank AND 1 fyke for 48 hours parallel with and on both riverbanks
Canalized rivers with too high conductivity for electrofishing (depth: < 1.30 m)	seine netting	100 m, two times complete seine netting
Lakes	electric fishing AND fykes	15% of riverbank (minimum 1000 m, maximum 2000 m) or 100% if perimeter is less than 1000 m AND 1 fyke/hectare (minimum 4, maximum 20 fykes)
Estuaries	fyke fishing, anchor netting, pound netting, electric fishing	Fykes: 2 paired nets for two successive days per site. Winged fyke: one per site for two successive days. Anchor netting: per site 4 surveys of one hour (two for each tide). Electric fishing: only in flood control areas (250 m shore transects/ha).

## Project data

### Project title

VIS – Fish Information System

### Personnel

**Principal investigator:** Hugo Verreycken, Jan Breine, Gerlinde Van Thuyne

**Resource contact, resource creator, metadata provider, point of contact:** Gerlinde Van Thuyne (Inland Waters), Jan Breine (Estuarine Waters)

**Content providers:** Daniel Bombaerts, Jan Breine, Jean-Pierre Croonen, Adinda De Bruyn, Franky Dens, Marc De Wit, Linde Galle, Isabel Lambeens, Yves Maes, Gerlinde Van Thuyne

**Developer:** Tom De Boeck

**Processors:** Dimitri Brosens, Peter Desmet

### Funding

Flemish government
